# Cyclodextrin Complexed Lipid Nanoparticles of Irbesartan for Oral Applications: Design, Development, and In Vitro Characterization

**DOI:** 10.3390/molecules26247538

**Published:** 2021-12-13

**Authors:** Narendar Dudhipala, Swetha Ettireddy, Ahmed Adel Ali Youssef, Goverdhan Puchchakayala

**Affiliations:** 1 Department of Pharmaceutics, Vaagdevi Pharmacy College, Warangal 506005, Telangana, India; goverdhanucpsc@gmail.com; 2 Synapse Life Sciences, Warangal 506001, Telangana, India; swethareddy532@gmail.com; 3 Department of Pharmaceutical Technology, Faculty of Pharmacy, Kafrelsheikh University, Kafrelsheikh 33516, Egypt; eldiasti89@pharm.kfs.edu.eg

**Keywords:** irbesartan, complexation, cyclodextrin, solid lipid nanoparticles, sustained release

## Abstract

Irbesartan (IR) is an angiotensin II receptor antagonist drug with antihypertensive activity. IR bioavailability is limited due to poor solubility and first-pass metabolism. The current investigation aimed to design, develop, and characterize the cyclodextrin(s) (CD) complexed IR (IR-CD) loaded solid lipid nanoparticles (IR-CD-SLNs) for enhanced solubility, sustained release behavior, and subsequently improved bioavailability through oral administration. Based on phase solubility studies, solid complexes were prepared by the coacervation followed by lyophilization method and characterized for drug content, inclusion efficiency, solubility, and in vitro dissolution. IR-CD inclusion complexes demonstrated enhancement of solubility and dissolution rate of IR. However, the dissolution efficiency was significantly increased with hydroxypropyl-βCD (HP-βCD) inclusion complex than beta-CD (βCD). SLNs were obtained by hot homogenization coupled with the ultrasonication method with IR/HP-βCD inclusion complex loaded into Dynasan 112 and glycerol monostearate (GMS). SLNs were evaluated for physicochemical characteristics, in vitro release, differential scanning calorimetry (DSC), powder X-ray diffractometry (PXRD), and physical stability at room temperature for two months. The optimized SLNs formulation showed particle size, polydispersity index, zeta potential, assay, and entrapment efficiency of 257.6 ± 5.1 nm, 0.21 ± 0.03, −30.5 ± 4.1 mV, 99.8 ± 2.5, and 93.7 ± 2.5%, respectively. IR-CD-SLN and IR-SLN dispersions showed sustained release of IR compared to the IR-CD inclusion complexes. DSC results complimented PXRD results by the absence of IR endothermic peak. Optimized IR-CD complex, IR-SLN, and IR-CD-SLN formulations were stable for two months at room temperature. Thus, the current IR oral formulation may exhibit improved oral bioavailability and prolonged antihypertensive activity, which may improve therapeutic outcomes in the treatment of hypertension and heart failure.

## 1. Introduction

Oral formulations account for around 90% of the global market share of all pharmaceutical formulations intended for human use. According to current estimates, orally administered pharmaceutical products account for about 84 percent of the best-selling pharmaceutical items, which are currently valued at $35 billion and growing at a 10% yearly pace [1,2]. Patient compliance to oral medications is generally higher than other routes of administration such as intravenous, subcutaneous, and intramuscular, as well as to inhalation medications. Despite, this main advantage, the development of oral formulations poses several challenges. Oral bioavailability (BA) of poorly water-soluble drugs and drugs prone for first-pass metabolism often is low as their absorption could be kinetically limited by low rates of dissolution and/or capacity-limited by poor solubility when these drugs were administered in traditional solid formulations. Problems such as poor solubility or chemical stability in the environment of the gastrointestinal tract (GIT), poor permeability through the biological membranes, or sensitivity to metabolism are well known to result in the rejection of potential drug candidates as practical products [3,4].

Various methodologies have been applied to enhance the oral BA of drugs with poor aqueous solubility [5,6,7]. Among various approaches, solid dispersions (SDs) are generally prepared using a hydrophilic polymer, and a drug with poor aqueous solubility by solvent evaporation, melting-extrusion, coprecipitation, or spray drying method to enhance the solubility and dissolution rate of poorly water-soluble drugs [8]. Formation of inclusion complex with drug molecules is another approach to increase the aqueous solubility of poorly water-soluble drugs, and it provides better control on the release rates of lipophilic drugs as well [9]. 

Lipid-based drug delivery systems are one of the emerging approaches, designed to address oral drug delivery challenges. Higher solubilization and absorption can be achieved by encapsulating or solubilizing drug molecules in lipid excipients, resulting in increased BA of the drug candidates. The use of lipid-based drug delivery systems such as SLNs have generated much academic and commercial interest as a potential formulation strategy for improving the oral BA and overcoming the hepatic first-pass metabolism. SLNs are used as an alternative carrier system to emulsions, liposomes, and polymeric micro and nanoparticles. SLNs are sub-micron spherical colloidal nanoparticles having a size range of 50–1000 nm with a drug-containing solid lipid core stabilized with surfactants [10]. SLNs enhance the BA of drug molecules by the uptake of the nanocarrier through the GIT directly, enhanced the permeability through the GI membrane, adhesion to the GI tract wall, and enhance intestinal lymphatic transport of the lipophilic drugs and therefore increase oral BA [3,10]. In addition, SLNs possess additional advantages; encapsulating both hydrophilic and lipophilic drugs, biocompatibility which is due to the biodegradable nature of lipids, controlled drug release rate, high drug content compared to other carrier systems, feasibility in scale-up, and suitability for other routes of administration such as oral, intravenous, pulmonary, ocular and transdermal drug administration [10,11,12].

Irbesartan (IR) is chemically 2-butyl-3-([2-{1 H-tetrazole-5-yl}biphenyl-4-yl]-1,3-diazaspiro (4,4)non-1-en-4-one, a non-peptide angiotensin II type 1 (AT_1_) receptor antagonist, used in the treatment of hypertension and heart failure [13,14]. The major drawback during therapeutic application of IR as an oral dosage form is its very low aqueous solubility (91.3 µg/mL) and first-pass metabolism [14,15]. According to Biopharmaceutical Classification System (BCS), IR is classified as a BCS class II drug (low aqueous solubility and high permeability).

Cyclodextrins (CDs) are widely used as excipients in pharmaceutical formulations. Inclusion complex formation using CDs with drug molecules came into existence as potential carriers to improve the oral delivery of lipophilic drugs [16]. CDs are cyclic oligosaccharides that are composed of glucopyranose units. The conical cylinder formed by the glucopyranose units has a hydrophobic interior cavity and a hydrophilic outer surface. Alpha, beta, and gamma CD (αCD, βCD, and γCD) and their modified derivatives such as hydroxypropyl-βCD (HP-βCD), randomly methylated-βCD, and sulfobutylether-βCD are the main family members that are chosen for inclusion complexation [1].

Previously, the solubility of IR was enhanced by complexation with βCD and HP-βCD [17,18]. IR: βCD inclusion complex (IR-βCD) was prepared with three different methods such as co-grinding, kneading, and co-evaporation methods. Among these methods, the co-evaporation method was considered as a suitable method, in which the significant improvement in solubility and dissolution rate of the IR was observed compared with other methods and pure IR. In another study, IR-loaded SLNs (IR-SLNs) were reported to improve the oral BA of IR by 1.4-folds compared with IR alone in Wistar rats [19]. However, IR-CD complexed nanoformulations have not been reported till now. Therefore, we attempted to develop IR-CD-loaded SLNs (IR-CD-SLNs) to enhance solubility, promote lymphatic transport to minimize first-pass metabolism, and sustain IR release through oral administration. The objective of the current investigation was to prepare and characterize the optimized IR-CD complex with the co-evaporation followed by lyophilization method. In addition, IR-CD-SLNs and IR-SLNs formulations were prepared and optimized based on physicochemical characteristics and in vitro release studies. Furthermore, stability, and crystalline nature of IR-CD, IR-SLN and IR-CD-SLN formulations were evaluated.

## 2. Materials and Methods

IR was a kind gifted sample from Lupin Research Park Ltd., Pune, India. βCDs (Molecular weight (MW), 1135) and HP-βCD (MW. 1500) were a generous gift from Dr. Reddy’s Labs Pvt Ltd. (Hyderabad, Telangana, India). Tween^®^ 80, Dynasan 112, and Poloxamer 188 were purchased from Sigma-Aldrich Chemicals (Bangalore, Karnataka, India). Glycerol monostearate (GMS) was purchased from RX CHEMICALS (Navi Mumbai, Maharashtra 400705, India). Soylecithin was a generous gift from Lipoid (Ludwigshafen am Rhein, Germany). Methanol, acetonitrile, chloroform, and ethanol were of HPLC grade (Merck, Bengaluru, Karnataka, India). Centrisart filters (M.W. cut off 20,000 kD) were purchased from Sartorius (August-Spindler-Straße 11, 37079 Göttingen, Germany).

### 2.1. Quantification of IR by HPLC

IR was quantified using a slightly modified reported HPLC method [20]. The HPLC system consists of LC-20 AD solvent delivery unit with reverse-phase Merck C18 (250 × 4.6 mm; 5 microns) (Lichrosphere, Merck, Germany) column, was equilibrated with an eluent mixture of phosphate buffer (pH 4)/acetonitrile (50:50, *v*/*v*) pumped isocratically at a flow rate of 1 mL/min. The peaks were obtained at 270 nm wavelength using an SPD-20AV ultraviolet detector (Shimadzu, Japan). All the samples were analyzed at 25 °C at absorbance units full scale (AUFS) 1.00 and 20 μL injection volume. Before the elution, the mobile phase was filtered (Millipore system, 0.2 μm) under vacuum and degassed. The total run time for the analysis was 5 min.

### 2.2. Phase Solubility Studies:

Phase solubility studies with increasing concentration of βCD and HP-βCD (0.1, 0.25, 1, 1.5, 2.5, 4, and 5 millimole ratio) were performed according to the method described by Higuchi and Connors [21]. Briefly, an excess amount of the drug was added to 10 mL of double-distilled water containing various concentrations of βCD and HP-βCD in a stoppered glass vial. The suspensions were vigorously shaken at 25 ± 1 °C for 72 h on a rotary shaker. After equilibrium was attained, the samples were filtered through a 0.45 µm Millipore membrane filter and suitably diluted. These samples were assayed for the drug content by UV spectrophotometer against blank in the same concentration of βCD or HP-βCD in water. The solubility experiments were conducted in triplicate. The IR-CD apparent stability constant K_1:1_ and the complex efficiency (CE) were calculated from the slope of the linear plot of the phase solubility diagram and the solubility of IR alone in water without the complexing agents according to Equations (1) and (2).

(1) $$K_{1:1}=\frac{\mathrm{Slope}}{S_{0}\left(1-\mathrm{Slope}\right)}$$

where S_0_ is drug solubility in the absence of βCD and HP-βCD (intercept).

(2) $$\mathrm{CE}=S_{0}\times K_{1:1}=\frac{\mathrm{Slope}}{1-\mathrm{Slope}}$$

### 2.3. Preparation of Physical Mixture

Physical mixtures (PM) of IR-βCD and IR-HP-βCD in the ratio of 1:1 were prepared by mixing of individual components (weighed amount of IR, βCD, and HP-βCD) that had been previously sieved through a mesh no. 80 sieve (180 μm) in a glass mortar until a homogeneous mixture was obtained [22]. Then, these mixtures were stored in the desiccators. This mixture was used for the comparative study with IR-βCD and IR-HP-βCD lyophilized products.

### 2.4. Preparation of SDs

Based on phase solubility graphs an AL-type means that the complexation ratio of CD: IR is on a 1:1 molar basis. Briefly, the CD was dissolved in distilled water to prepare CD solution (1 mM), and a 30% methanolic solution containing IR was prepared. The methanolic solution of IR was dropped stepwise to the aqueous solution of CDs. The mixture was stirred at 600 rpm for 6 h until a stable suspension was formed on a magnetic stirrer to obtain complexation equilibrium. The suspension was further sonicated. Then, the clear solution was kept on a magnetic stirrer until all solvent molecules were evaporated. All the clear solutions were frozen at −20 °C and the frozen mass was lyophilized in a freeze-dryer (Lyodel, Delvac Pumps Pvt. Ltd., Kanchipuram, Tamil Nadu, India) for 72 h to obtain a dry powder of IR-βCD and IR-HP-βCD SD formulations.

### 2.5. Drug Content

The drug content of SDs and PM was assayed to calculate the equivalent amount of IR to be used. The drug content in the prepared SD and PM formulations was analyzed by taking an appropriate weighed quantity (in triplicate), dissolving in 25 mL of methanol in a conical flask, and keeping in a rotary shaker for 1 h. Then, samples were centrifuged at 15,000 rpm for 15 min and the collected supernatant was filtered through a 0.45 µm membrane filter, properly diluted, and analyzed spectrophotometrically at a UV detection λ of 220 nm (SL-159, Elico Ltd., Hydrabad, Telangana 500018, India).

### 2.6. Inclusion Efficiency

The prepared SDs and PMs were dissolved in 10 mL methanol in 10 mL volumetric flasks, mixed thoroughly, and sonicated for 30 min. After proper dilution with methanol, the solution was analyzed spectrophotometrically at a UV detection λ of 220 nm.

### 2.7. In Vitro Dissolution Study

SDs, PMs, and pure IR were subjected to in vitro dissolution test to ascertain the role of CDs in enhancing the dissolution behavior of hydrophobic drugs. The test was performed in triplicate using USP type II apparatus (Paddle) (TDT-06P, Eletrolab, Goregaon East, Mumbai, Maharashtra 400063, India) in phosphate buffer (pH 6.8, 37 ± 0.5 °C, 50 rpm) as the dissolution medium. Relevant quantities of formulations (SDs and PMs) and IR equivalent to 5 mg dose were placed into the dissolution media. Aliquots of 5 mL were withdrawn at predetermined time intervals and replaced with an equal volume of fresh medium. Samples were analyzed using a UV spectrophotometer.

### 2.8. Dissolution Studies

The percentage cumulative amount of IR released in 15 and 60 min (Q 15 and Q 60 respectively) were derived from the in vitro dissolution release profile. Data were processed using the statistical function of Microsoft365^®^ office excel (2019, Microsoft Corporation, Redmond, WA, USA).

### 2.9. Dissolution Efficiency (DE)

DE is defined as the area under the dissolution curve up to a certain time (t), expressed as a percentage of the area of the rectangle described by 100% dissolution at the same time. DE was calculated by using Equation (3):

(3) $$\%\mathrm{DE}=\frac{\int_{0}^{t}y\times \mathrm{dt}}{y_{100}\times t}\times 100$$

where y is the percentage of dissolved product, t is time, and y_100_ is expressed as a percentage of the curve at maximum dissolution.

#### 2.9.1. Mean Dissolution Rate (MDR)

The sum of the drug coming into the bulk of dissolution media per unit time under in vitro dissolution conditions is defined as the MDR. MDR was calculated by using Equation (4):

(4) $$\mathrm{MDR}=\frac{\sum_{j=1}^{n}∆M_{j}/∆t}{n}$$

where ΔM_j_ is the additional amount of drug dissolved between t_j_ and t_1_, n is the number of in vitro dissolution sample times, Δt is the midpoint time between t_j_ and t_1_ which can be easily calculated with (t + t_1_)/2.

#### 2.9.2. Mean Dissolution Time (MDT)

MDT was calculated to assess the comparative extent of the dissolution rate enhancement from CDs. It is defined as the time taken for dissolving a molecule from a solid dosage. MDT was calculated according to Equation (5):

(5) $${\mathrm{MDT}}_{\text{in}\;\text{vitro}}=\frac{\sum_{i=1}^{n}t_{\mathrm{mid}}\cdot ∆M}{\sum_{i=1}^{n}∆M}$$

where i is the in vitro dissolution sample number, n defines the number of sample time points, t_mid_ is the midpoint of the ith period easily calculated with [t + t_1_)/2 and ΔM is the additional amount of drug dissolved between t_i_ and t_1_.

#### 2.9.3. Initial Dissolution Rate (IDR)

IDR is the first 15 min dissolution rate during the in vitro dissolution study and is called the dissolution rate.

### 2.10. Preparation of IR-SLNs and IR-CD-SLNs

SLNs were prepared by the hot homogenization coupled with the ultrasonication method [23]. Briefly, IR, solid lipid (GMS or Dynasan 112), and soylecithin were dissolved in 5 mL of 1:1 chloroform and methanol mixture for SLNs preparation. Organic solvents were removed completely using a rotary evaporator (Heidolph, Schwabach, Germany). The drug-containing oily phase layer was melted by heating above the melting point of the solid lipid by 5 °C. However, during the preparation of IR-CD-SLNs, IR was added as an inclusion complex to the aqueous phase. An aqueous phase consisting of IR-CD complex (52.6 mg) and/or Poloxamer 188 (1.5% *w*/*v*) was dissolved in double-distilled water to prepare the aqueous phase and heated to the same temperature as the oily phase. The hot aqueous phase was added dropwise to the oily phase under continuous magnetic stirring at 2000 rpm for 5 min and homogenized at 16,000 rpm using a high-speed homogenizer (Diax900, Heidolph, Germany) for another 5 min. The obtained O/W emulsion was ultrasonicated using a 12 T probe Sonicator (Vibracell, Sonics, CT, USA) for 25 min at 40% amplitude with a 10-sec pulse ON and 10-sec pulse OFF. SLNs were obtained by allowing hot nanoemulsion to cool at room temperature. The drug load of IR in SLNs was maintained at 0.1% *w*/*v* (1 mg/mL).

### 2.11. Preparation of IR Suspension (IR-CS)

IR-CS was prepared by dissolving 0.1% *w*/*v* of IR in double-distilled water by using 1% *w*/*v* Tween^®^ 80 as a suspending agent. The mixture was stirred for 1 h at 2000 rpm at 40 °C, followed by 1 h at room temperature.

### 2.12. Characterization of IR-SLNs and IR-CD-SLNs

#### 2.12.1. Particle Size (PS), Polydispersity Index (PDI), and Zeta Potential (ZP)

The PS, PDI, and ZP of SLNs were measured using a Malvern Zetasizer (Nano ZS90, Malvern Panalytical Ltd, Enigma Business Park, Grovewood Rd, Malvern WR14 1XZ, UK). For the measurement of PS and PDI, the prepared SLNs of 100 µL were diluted to 5 mL with double distilled water to get optimum Kilo Counts Per Second (KCPS) of above 100 for measurements. ZP of the SLN formulations was measured using the electrode cell based on the Smoluchowski equation using the same diluted sample. All analyses were carried out in triplicate at 25 °C [24].

#### 2.12.2. Drug Content

About 100 µL of the IR-loaded SLNs was dissolved in a 900 µL mixture of chloroform and methanol (1:1), vortexed for 5 min, and centrifuged at 15,000 rpm for 15 min (Remi Laboratory Instruments, Cama Industrial Estate, Goregaon, Mumbai, Maharashtra 400063, India). The samples were further diluted with the mobile phase before injection into the HPLC column [25]. The amount of IR in SLN formulations was quantified using the HPLC method mentioned above. The IR content (assay) was used to calculate the percentage entrapment efficiency of IR in SLNs.

#### 2.12.3. Entrapment Efficiency (EE)

EE was determined by measuring the concentration of the free drug (unentrapped) in the external aqueous phase based on an earlier reported method [26]. The aqueous medium was separated by ultra-filtration using centrisart tubes (Sartorius, Goettingen, Germany), which consisted of a filter membrane (Mwt. cut off 20 KDa) at the base of the sample recovery chamber. About 2.5 mL of the SLNs were placed in the outer chamber, and the sample recovery chamber was placed on top of the sample and centrifuged at 15,000 rpm for 15 min. The SLNs, along with the encapsulated drug, remained in the outer chamber and the aqueous phase moved into the sample recovery chamber through the filter membrane. The amount of IR in the aqueous phase was estimated using the above described HPLC method. The EE of the IR-LNFs were calculated using the following equation:

$$\%\mathrm{EE}=\left[\frac{\mathrm{Amount}\;\mathrm{of}\;\mathrm{IR}\;\mathrm{in}\;\mathrm{assay}-\mathrm{Amount}\;\mathrm{of}\;\mathrm{the}\;\mathrm{unentrapped}\;\mathrm{IR}}{\mathrm{Amount}\;\mathrm{of}\;\mathrm{weighed}\;\mathrm{IR}}\right]\times 100$$

#### 2.12.4. In Vitro Release Studies

The dialysis method was performed for in vitro release testing using Franz diffusion cells. Dialysis membrane (Hi-Media, Mumbai, India) having pore size 2.4 nm and MW cut-off between 12,000–14,000 kD was used for the release studies [27]. The dialysis membrane was soaked overnight in double distilled water before the release studies. Phosphate buffer (pH 7.4) with 40% ethanol was used as the release medium [27]. According to USP, organic cosolvents such as ethanol are routinely added as solubility modifiers to the aqueous dissolution media to afford sink conditions. The experimental unit was consisting of a donor and receiver compartment. The donor compartment contained 0.5 mL of the SLN formulation, and the receiver compartment had 12 mL of a freshly prepared release medium. The diffusion cell temperature was maintained at 37 ± 0.5 °C. At 0.5, 1, 2, 3, 4, 6, 8, 10, 12, 24, and 48 h time points, a 1 mL sample was withdrawn from the receiver compartment and replaced with an equal volume of the fresh receiver medium. The collected samples were analyzed for IR using HPLC to obtain the % cumulative amount released vs time profile.

#### 2.12.5. Lyophilization

The optimized IR-SLN and IR-CD-SLN formulation containing trehalose dihydrate as a cryoprotectant (10% *w*/*w*) were lyophilized in a freeze dryer (Lyodel, Delvac Pumps Pvt. Ltd., Tamil Nadu, India) to form a dry and free-flowing powder. The vacuum was applied, and the frozen samples were subjected to various drying phases for about 24 h to get the lyophilized SLNs product [28]. Lyophilized SLNs were reconstituted and used for further studies. The frozen samples were obtained by keeping the samples overnight in an ultra-freezer at −80 °C (Sanyo Electric Co., Ltd., Moriguchi, Osaka prefecture, Japan).

### 2.13. Solid-State Characterization

#### 2.13.1. Differential Scanning Calorimetry (DSC)

DSC is a thermodynamical tool for the assessment of the heat energy uptake under a regulated increase or decrease in temperature that occurs within a sample. DSC is one of the most common techniques for determining drug-excipient compatibility and the crystalline behavior of the drug and other different excipients. DSC thermograms of pure IR, pure CD, pure solid lipid, PM of IR, and solid lipid (1:1), freeze-dried IR-CD inclusion complex (1:1), lyophilized IR-SLNs, and lyophilized IR-CD-SLNs were obtained by DSC 822 e/200 (Mettler-Toledo (Schweiz) GmbH, Im Langacher 44, 8606 Greifensee Schweiz, Switzerland) instrument. Enthalpy changes during endothermic or exothermic changes were quantified. The instrument was calibrated with indium (calibration standard, purity > 99.99%) before the analysis. About 8 mg sample was used for analysis into a standard aluminum pan while an empty pan was used as a reference. The heating rate was increased at the rate of 10 °C/min and the obtained thermograms were observed for crystallinity changes and compatibility of IR with other excipients.

#### 2.13.2. Powder X-ray Diffraction (PXRD)

XRD technique was used to assess the polymorphic transitions (if any) of IR loaded into CD inclusion complex and SLNs formulations. XRD patterns of samples were recorded on a powder X-ray diffractometer (XRD 6000, SHIMADZU CORPORATION, 3, Kanda-nishiki-cho 1-chome Chiyoda-ku, Tokyo 101-8448, Japan), operating in the θ–2θ mode. The radiation originated from the source (40 kV and 30 mA) goes through a nickel filtered CuKα. Diffraction data were collected over a range of angles from 2 °C to 50°, at a step size of 0.045° and step time of 0.5 s, with an overall scan time of approximately 50 min. The samples were mounted on a glass sample holder. Diffraction patterns were simulated from published single-crystal X-ray diffraction data. Samples used for XRD analysis were pure IR, pure solid lipid, physical mixture of solid lipid and IR, and lyophilized IR-CD inclusion complex and IR-CD-SLNs formulations.

#### 2.13.3. Stability Studies of IR-CD, IR-SLN, and IR-CD-SLN

Optimized IR-CD inclusion complex, IR-SLN, and IR-CD-SLN formulations were subjected to stability studies by the storage of the formulations at room temperature (RT). Formulations were packed in 20-mL-capacity scintillation glass vails and kept at 25 ± 2 °C for 60 days. Samples were withdrawn at predetermined time intervals and analyzed [29]. Changes in parameters such as drug content and inclusion efficiency and PS, PDI, ZP, assay, and EE of IR-CD complex and IR-SLN, IR-CD-SLN, respectively were recorded.

#### 2.13.4. Statistical Analysis

The statistical significance comparison of data was made with an unpaired Student’s *t*-test using GraphPad prism software (version 5.02, 2013, San Diego, CA, USA). A statistically significant difference was observed at a ‘*p*’ value less than 0.05 (*p* < 0.05).

## 3. Results and Discussion

### 3.1. Phase Solubility Studies

The ability of a CD to form an inclusion complex is a function of steric as well as thermodynamic factors [30]. The driving force leading to inclusion complexation involves the exclusion of hydrophobic cavity-bound high-energy water and formation of Van der Waals forces, hydrophobic, electrostatic, and hydrogen bond interactions as well as charge–transfer interaction [30]. CDs can form inclusion complexes with low aqueous solubility drugs and change the physicochemical and biological properties of guest molecules due to their unique structure [31,32]. CDs act as permeation enhancers by carrying the drug through the aqueous barrier, from the bulk solution inside the GI tract to the lipophilic surface of biological membranes, where the drug molecules can partition easily from the inclusion complex into the lipophilic membrane [30]. Moreover, CDs have the added other advantage of improving the chemical stability of drug molecules, especially esters, by protecting chemically labile substances from potentially tough environmental conditions and reducing also the enzymatic degradation, oxidation, or hydrolysis [1].

**Figure 1:**
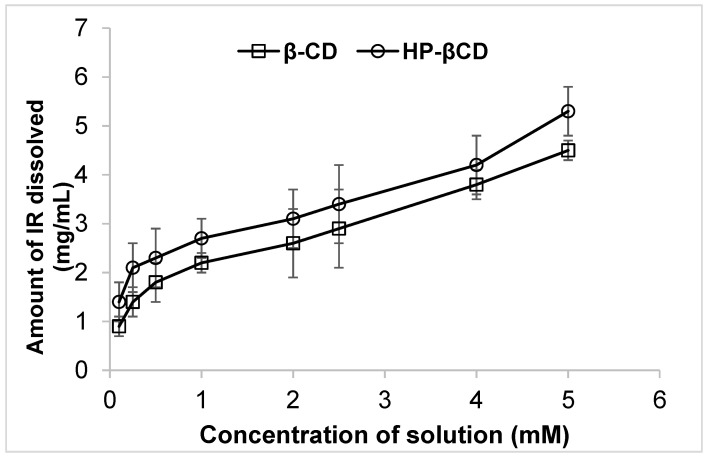
Phase solubility diagram of IR–βCD and IR-HP-βCD inclusion complex in water at 25 ± 2 °C (mean ± SD, *n* = 4).

The phase solubility profiles of IR–βCD and IR-HP-βCD are presented in Figure 1. The solubility of IR was increased with the increasing concentration of βCD and HP-βCD; therefore, this phase solubility diagram could be classified as A_L_ type according to Higuchi and Connors [22,33]. A complex with a 1:1 molar ratio was formed, according to the linear host–guest correlation (correlation coefficient R^2^ 0.97 and 0.96 for βCD and HP-βCD respectively). Furthermore, K_1:1_ was 642.7 M^−1^ and 491.6 M^−1^ for IR–βCD and IR-HP-βCD, respectively. The reported value of K_1:1_ is most often between 50 and 2000 M^−1^ with a reported mean value of 490 M^−1^ for the parent βCD [34,35]. However, the determined K_1:1_ value is strongly affected by the intercept. The feasibility of using CDs as pharmaceutical excipients can be calculated from K_1:1_ and S_0_ [34]. The results came in accordance with Hirlekar et al. and Leoins et al. [17,22].

### 3.2. Drug Content and Inclusion Efficiency

The CE of the CDs in the aqueous vehicle is the key aspect and not the absolute value of K_1:1_. S_0_ is sharply affected by many pharmaceutical excipients such as preservatives, buffer salts, and polymers, and sometimes S_0_ is below the detection limit of the analytical method used for the quantitative determination of the drug. Since the numerical value of CE is dependent only on the slope of the phase-solubility profile; therefore, less variation is usually observed in the value of CE compared to the K_1:1_ value. The obtained results are summarized in Table 1. The % complexation efficiency was 93.9 ± 2.2—75.2 ± 2.3 for IR–βCD inclusion complex, compared to 95.6 ± 3.1—80.6 ± 2.2 for IR-HP-βCD inclusion complex. The relative standard deviation (RSD) values in the limits of ±5% indicate that there are no significant changes by the sample processing. IR: βCD (1:4) and IR: HP-βCD (1:4) showed the best results regarding drug content, inclusion efficiency, and folds improvement in IR solubility. Therefore, these two formulations have been considered as the optimized formulations for further dissolution studies.

### 3.3. Analysis of Dissolution Data

**Figure 2:**
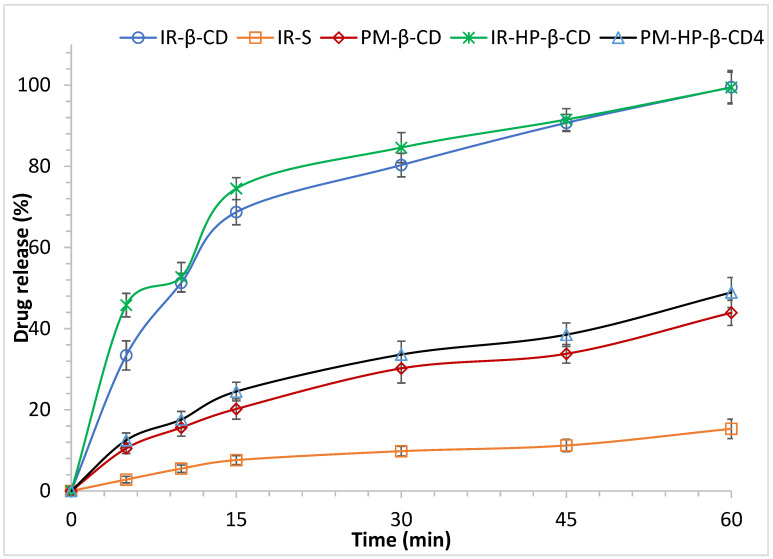
The in vitro release profile of irbesartan from irbesartan solution, PMs, and the corresponding cyclodextrin inclusion complexes in phosphate buffer (Mean ± SD, *n* = 4).

The dissolution profiles of pure IR, PMs and the IR-CDs inclusion complexes are depicted in Figure 2. The release rate profiles were plotted as the percentage of IR released vs time. According to these results, the inclusion complexes released up to 76.2 and 94.4% of the drug in 60 min from IR-βCD and IR-HP-βCD, respectively, whereas the pure drug exhibited the release of ~7.6% after 15 min and not more than 15.3% at 60 min. However, PM of IR/βCD and PM of IR/HP-βCD released up to 40.7 and 43.9% of the drug in 60 min, respectively. These quantities contrast with the markedly 4.9- and 6.2-fold increase in the release of IR-βCD and IR-HP-βCD, respectively compared to the pure drug. Additionally, the results showed a marked 1.9- and 2.2-fold increase in the release of IR-βCD and IR-HP-βCD, respectively, compared to the corresponding PMs. DE values at 15 and 60 min are shown in Table 2. DE are suitable parameters for the evaluation and comparison of the in vitro dissolution of different formulations. This parameter is defined as the area under the dissolution curve (AUC) up to a specific time, t, expressed as the rectangle described by 100% dissolution in the same time [36]. The DE (60 min) of the IR-βCD formulation is 2.8- and 1.9-fold greater as compared to the drug alone and the corresponding physical mixture, respectively. However, the DE (60 min) of the IR-HP-βCD formulation is 3.4- and 1.4-fold greater as compared to the drug alone and the corresponding physical mixture, respectively. All the formulations containing CDs showed a significant difference in the DE with respect to IR (*p* < 0.05). The maximum dissolution of active pharmaceutical ingredients from a dosage form in a particular period of time is not the only standard related to drug absorption, despite the pharmacopoeial test methods would show otherwise. The dissolution rate is also critical and is frequently the rate-limiting process for drug absorption [37]. A calculation of the rate of dissolution would necessitate the development of a suitable kinetic law. However, the complex agent dissolution and drug dissolution, overlap, and such a technique would have to be approached with caution. MDT is used as a model-independent approach. The MDT reflects the dissolution rate and the shorter MDT, the faster the progress of the dissolution process. The values for MDT are shown in Table 2. PMs showed an insignificant difference in MDT when compared to the pure drug (*p* > 0.05); while, the corresponding inclusion complexes showed a significant difference in MDT (*p* < 0.05), compared to the pure IR. CDs are useful excipients that have widespread attention and use in pharmaceutics and drug delivery. The reason for this popularity is the ability of these excipients to interact with lipophilic drug candidates resulting in an increase in the apparent water solubility of these drug candidates. In this regard, this solubilization mechanism is due to the ability of CDs to form non-covalent dynamic inclusion complexes in the solution. Moreover, the solubilizing mechanisms may include the formation of a non-inclusion-based complex, the formation of aggregates and related domains, and the ability of CDs to form and stabilize supersaturated drug solutions [38,39,40]. The increase in IR solubility also can increase the dissolution rate and thus could improve the oral bioavailability of IR [39]. Based on the dissolution studies and its parameters, IR/HP-βCD inclusion complex was selected to be loaded in SLNs formulations.

### 3.4. IR-SLNs and IR-CD-SLNs

Four different SLN formulations were prepared using Dynasan 112 and GSM, selected based on earlier reported studies [19]. The composition of all prepared SLN formulations is shown in Table 3. Surfactants (Poloxamer 188 and Soyalecithin) were used to prepare the aqueous phase. Poloxamer 188 is a non-ionic surfactant, added to the lipid nanoformulations as a steric stabilizer, to promotes electrostatic stabilization because it decreases the electrostatic repulsion between the nanoparticles by creating a coat around the surface for keeping the stability of lipid nanoparticles [29,41].

### 3.5. PS, PDI, and ZP of IR-SLNs and IR-CD-SLNs

Uptake and distribution of lipid-based nanoparticles by the lymphatic system depend on many factors such as molecular weight, type of lipid, PS, surface charge, surfactant concentration, and hydrophobicity [42]. Avoiding first-pass metabolism in the liver and GIT is the major advantage in drug delivery through uptake by the lymphatic system. The small PS of these formulations (20–500 nm) enables efficient uptake of drug molecules into the intestine, particularly via the lymphatic route [42,43]. PS, PDI, and ZP are depicted in Table 4. A homogeneous system with PS ranging from 240.2 ± 6.7 to 339.9 ± 8.3 nm, with a surface charge from −20.5 ± 4.1 to −30.5 ± 4.1 mV and PDI from 0.19 ± 0.05 to 0.29 ± 0.08 were obtained with 3.5% *w*/*v* concentration of surfactant. The prepared batches of SLNs, containing GMS showed a significant increase in PS compared to SLNs that contain Dynasan 112 as the solid lipid (*p* < 0.05). However, the prepared batches of SLNs, containing Dynasan 112 showed a significant increase in ZP compared to SLNs that contain GMS (*p* < 0.05). Dynasan 112-based SLN dispersions showed a significantly lower PS than GMS-based SLN dispersions which could be due to the short hydrocarbon chain in the chemical structure of Dynasan 112 (Dodecanoic acid-based backbone) compared to the hydrocarbon chain in GMS (stearic acid-based backbone). However, GMS-based SLN dispersions showed a significantly lower ZP than Dynasan 112-based SLN dispersions which could be due to the fewer number of negatively charged carboxylic groups in the chemical structure of GMS (1 COO-) compared to the number of carboxylic groups in the chemical structure of Dynasan 112 (3 COO-). Nanoparticle formulations with PDI value ≤ 0.1 is considered as highly monodisperse, while PDI value >0.4 is considered highly polydisperse and value in range of 0.1–0.4 is considered to have a moderately disperse distribution [44].

### 3.6. Assay and EE of IR-SLNs and IR-CD-SLNs

EE and drug content in SLNs formulations were estimated by an HPLC and depicted in Table 4. EE ranged from 86.9 ± 1.8 to 93.7 ± 2.5%. Formulations containing Dynasan-112 showed slightly high values (90.6 ± 4.3% and 93.7 ± 2.5% for IR-SLN2 and IR-HP-βCD-SLN2) when compared to that of GMS (89.1 ± 2.8% and 86.9 ± 1.8% for IR-SLN1and IR-HP-βCD-SLN1), which is statistically significant (*p* < 0.05) when IR-HP-βCD-SLN1 is compared to IR-HP-βCD-SLN2. SLN formulations prepared with Dynasan-112 as the lipid matrix resulted in higher drug entrapment than GMS. Dynasan-112 has a C12 triglyceride chain that could produce less ordered lipid crystals than GMS which is composed only of a monoester of stearic acid. This observation came in accordance with earlier reported studies [45]. The imperfection of the lipid structure could offer more void space to accommodate more drug molecules that is beneficial to increase drug loading capacity [45,46]. EE is also dependent on the amount of lipid, drug solubility in the lipid melt, and the concentration of surfactant [45]. Since the loaded drug molecules are located between the fatty acid chains of the lipid, between the lipid layers, and also in crystal imperfections, a highly ordered crystal lattice does not offer accommodation large amounts of drug molecules and consequently lead to drug expulsion during storage [11,45]. The drug content in the SLN dispersions ranged from 98.4 ± 1.8 to 99.8 ± 2.5%.

### 3.7. In Vitro Release Studies of IR-SLNs and IR-CD-SLNs

**Figure 3:**
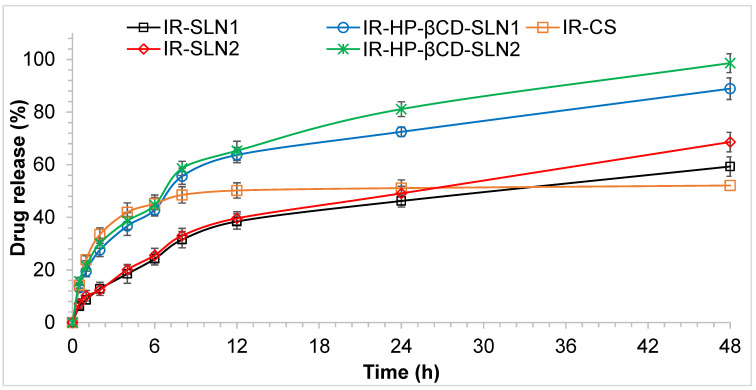
The in vitro release profile of irbesartan from IR-CS, IR-SLN, and IR-CD-SLN (mean ± SD, *n* = 4).

Based on the earlier reported solubility studies of IR in different dissolution media [47], Phosphate buffer (pH 7.4) with 40% ethanol was used as the receiver medium for in vitro release studies for 48 h by the dialysis method. The results are shown in Figure 3. During the *in vitro* release studies, a sustained release behavior of IR was observed. SLNs formulations containing Dynasan 112, and GMS showed drug release ranging from 65.9 ± 0.2 to 100.0 ± 1.5% (IR-SLN2 and IR-HP-βCD-SLN2) and 55.5 ± 1.1 to 85.4 ± 1.6% (IR-SLN1 and IR-HP-βCD-SLN1). The IR suspension showed a quite fast release rate in the first 4 h because the fraction of free IR in solution was immediately ready to diffuse but rapidly reached a pseudo plateau phase during the next 44 h, giving rise to a maximum of 45.2 ± 1.0% drug released, whereas the IR-SLNs exhibited relatively high drug release 55.5 ± 1.1% and 65.9 ± 0.2% for IR-SLN1and IR-SLN2, respectively. IR is a lipophilic drug that belongs to the BCS class-II, and this could be a reason for the sustained release behavior from the lipid matrix of SLNs formulations. However, IR-CD-SLNs exhibited high drug release 85.4 ± 1.6% and 100.0 ± 1.5% for IR-HP-βCD-SLN1and IR-HP-βCD-SLN2. Loading of IR in combination with HP-βCD, both as Dynasan 112 or GMS formulations, improved the IR release rate from both SLNs formulations, compared to the IR suspension and the corresponding SLNSs containing IR alone. The enhancement of IR release rate exhibited by the two formulations containing IR/HP-βCD inclusion complex could be due to the wetting and solubilizing effect of HP-βCD towards IR [48]. Moreover, the increase in drug release rate was more evident in the case of SLNs, containing Dynasan 112, giving rise to a high % drug release (100.02 ± 1.50%), where this observation could be due to the formation of the supercooled melts with an amorphous nature at room temperature. Based on literature review, it was observed that Dynasan-based nanoparticles could either exist into the solid crystalline state or the supercooled liquid state at room temperature. Supercooled melt behaves like O/W emulsions (liquid state), indicating that the mobility of drug molecules from supercooled melts is expected to occur easily [49]. Formulation containing Dynasan-112 (IR-SLN2 and IR-HP-βCD-SLN2) showed a small PS (240.2 ± 6.7 and 257.6 ± 5.1 nm), better PDI (0.19 ± 0.05 and 0.21 ± 0.03), a good value for ZP (−29.4 ± 4.8 and −30.5 ± 4.1 mV), high % EE (90.6 ± 4.3 and 93.7 ± 2.5), and release of 65.9 ± 0.2 and 100.0 ± 1.5%, respectively in a period of 48 h when compared to other formulations. Therefore, these two formulations were selected as the optimized formulations and used for further studies.

### 3.8. Stability Studies of the Optimized SLNs Formulation

Stability studies were conducted for finally optimized formulation (IR-βCD, IR-HP-βCD, IR-SLN2, and HP-βCD-SLN2) at RT for two months. The two inclusion complexes showed no significant (*p* > 0.05) changes in drug content and inclusion efficiency. Some changes were observed in ZP, PDI, assay, % EE, and PS values, but statistically insignificant as shown in Table 5.

### 3.9. Lyophilization

The transformation from liquid form to solid form could offer possibilities for incorporating SLNs into pellets, capsules, and tablets [45]. Furthermore, freeze-drying of SLN dispersions yields dry products that can be conveniently stored and reconstituted before use by adding an aqueous medium. Lyophilization could increase the stability of SLN dispersions for a longer period because the aqueous SLNs formulations are liable to physicochemical stability problems [45]. High sublimation velocities and high specific surface areas were reported for the diluted dispersions of SLNs [45]. Therefore, trehalose was required as a cryoprotectant to ensure the ease of redispersion of the freeze-dried powder without any aggregation [45]. The cryoprotectant forms a kind of ‘pseudo hydration shell’ by the interaction with the hydrophilic head groups of the surfactant molecules [45]. Freeze-dried SLNs powder was diluted (1 to 50) with filtered double distilled water. The behavior during freeze-drying of SLNs dispersion was studied with respect to PS, PDI, and ZP, and the data is shown in Table 6. A significant increase in PS and PDI was observed (*p* < 0.05). However, an insignificant change in ZP was observed (*p* > 0.05). The freeze-drying process could modify the PS and the shape of the SLNs [50]. The increase in PS and PDI is due to nanoparticles aggregation due to lyophilization. The observation was consistent with many earlier reported studies [45,51,52].

### 3.10. DSC

**Figure 4:**
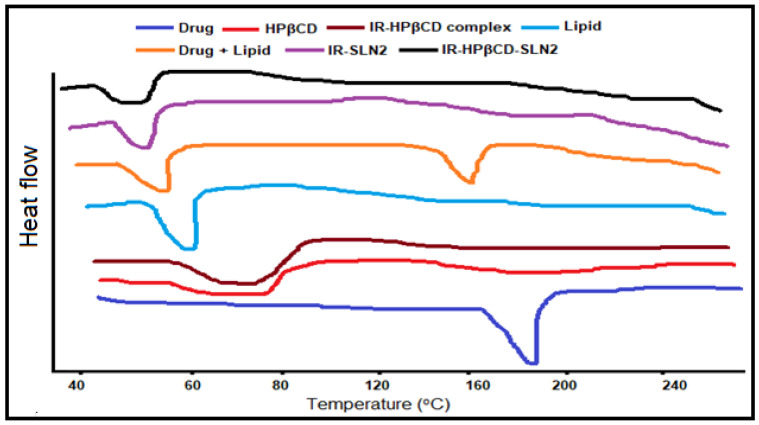
DSC curves of IR, HP-βCD, freeze-dried IR/HP-βCD inclusion complex (1:1 molar ratio), Dynasan 112, PM of IR, and Dynasan 112, freeze-dried IR-SLN2, and freeze-dried IR-HP-βCD-SLN2 formulations.

Thermal analysis techniques, mainly DSC, are broadly used in the pharmaceutical formulation field, starting from the quality control of raw materials to stability, and preformulation studies for the development of new formulations. DSC thermograms were used to determine the compatibility status of the drug, CDs, and lipids used in the preparation of SLN and CD-SLN formulations. Figure 4 shows the DSC thermograms of pure IR, pure HP-βCD, pure Dynasan 112, PM of drug and lipid (1:1), freeze-dried IR/HP-βCD inclusion complex (1:1), lyophilized IR-SLN2, and lyophilized IR-HP-βCD-SLN2 formulations. The DSC thermogram of pure IR and Dynasan-112 showed endothermic peaks at 185 °C and 48.56 °C, respectively [45,53]. The thermal curve of HP-βCD showed a broad endothermic peak at 90.11 °C corresponding to the dehydration and decomposition of HP-βCD [54]. The disappearance of the endothermic peak that corresponds to the melting point of pure IR was observed upon performing a similar DSC analysis of the freeze-dried inclusion complex. This observation confirms the formation of the inclusion complex of IR with HP-βCD. PM (1:1) of IR with Dynasan 112 showed IR peak at 200 °C; however, with less enthalpy and the endothermic peak of the lipid was slightly shifted to a lower temperature (47.82 °C). In general, the drug endotherm was well preserved. The absence of endothermic peak of IR in lyophilized IR-SLN2 and IR-βCD-SLN2 formulations indicated the conversion from crystalline to the amorphous form of IR molecules. The DSC curves did not show the characteristic IR peaks in SLN formulations due to the dispersion of the IR within the lipid matrix; the observed peaks are due to the solid lipid. The phase transition temperatures of colloidal dispersions were always significantly lower than those of anhydrous lipid mixtures [45]. In the case of colloidal SLN and CD-SLN dispersion systems, about a 5–10 °C decrease in the melting point of the lipid was observed. The phase transition temperature and enthalpy of the SLN formulation were much lower than those of the corresponding anhydrous physical mixture, as previously reported [45,55,56].

### 3.11. PXRD

**Figure 5:**
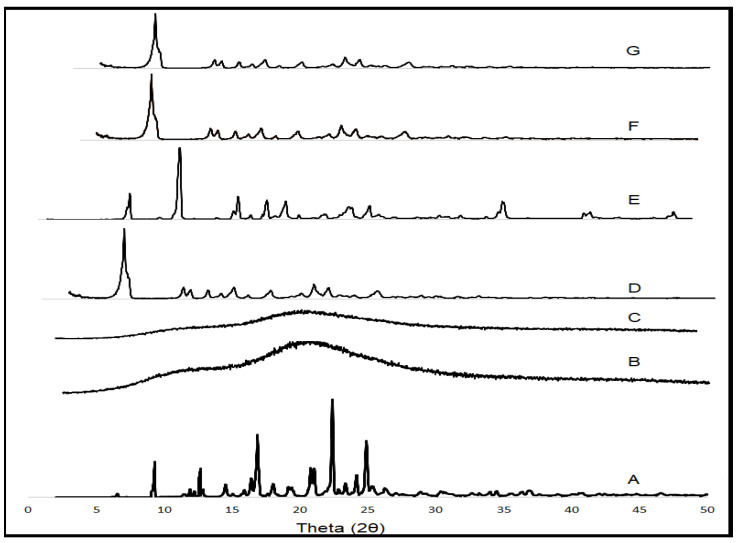
X-ray diffraction patterns of (A) IR, (B) HP-βCD, (C) IR-HP-βCD inclusion complex (1:1 molar ratio), (D) Dynasan 112, (E) IR + Dynasan 112 physical mixture, (F) lyophilized IR-SLN2 formulation, and (G) lyophilized IR-HP-βCD-SLN2 formulation.

XRD is a useful instrumental tool in the analysis, examination, monitoring of the crystal morphology of different drugs and excipients, used in drug formulation. Any changes that occurred in the crystalline state of drugs and excipients in the final product or formulation can be detected. XRD patterns of IR, HP-βCD, IR-HP-βCD inclusion complex (1:1 molar ratio), Dynasan 112, IR-Dynasan 112 PM, freeze-dried IR-SLN2, and IR-HP-βCD-SLN2 are shown in Figure 5. The XRD pattern of pure IR exhibits intense peaks at 2θ angles of 9.35°, 12.41°, 17.92°, and 23.05° which reveals the crystallinity characteristic of the drug [57]. The characteristic peaks of IR existed in the IR/Dynasan 112 PM. HP-βCD demonstrated only one broad peak centered at 20.5° which is consistent with its amorphous state. The XRD patterns of the freeze-dried IR/HP-βCD inclusion complex exhibited typical halo patterns for its amorphous structure, showing IR might be fully included in the cavity of HP-βCD [58]. The disappearance of IR peaks in the freeze-dried CD-SLN formulation indicated that IR was not existed in the crystalline state due to lyophilization [45]. Furthermore, the lyophilized formulation showed a decrease in the intensity of the characteristic lipid peaks and demonstrated the decreased crystallinity of the lipid. The XRD pattern did not also show the characteristic IR peaks in the lyophilized SLNs formulation due to the dispersion of the IR within the lipid matrix. X-ray diffraction data came in accordance with the DSC studies [59].

## 4. Conclusions

This study reports the successful preparation and optimization of a formulation based on the loading of IR-CD inclusion complex into SLNs for developing an oral liquid formulation of IR with enhanced solubility, sustained release profile, and could improve therapeutic efficacy. Of the tested CDs, HP-βCD was chosen based on its better performance in the in vitro release studies. The optimized IR-CD-SLNs were found to have small PS, narrow PDI, and a high entrapment efficiency with minimum stability of 2 months at room temperature. The two SLN formulations containing IR/HP-βCD inclusion complex exhibited enhancement of the drug release rate and sustained the drug release also. The optimized nanodispersions were lyophilized to improve the stability. However, morphology studies by scanning and transmission electron microscopy, long term stability studies at refrigerated and room temperature storage conditions, and ex vivo and in vivo pharmacokinetic studies are additional studies that are required for the formulation to be developed into an oral dosage form for the treatment of hypertension and heart failure. Thus, the present study suggests the potential development of nanoparticulate formulations for oral delivery of IR for the treatment of hypertension and heart failure.

## Figures and Tables

**Table 1:** Drug content and inclusion efficiency of irbesartan from PMs, IR-βCD, and IR-HP-βCD inclusion complexes (mean ± SD, *n* = 4).

Formulations	Drug Content (%)	Inclusion Efficiency (%)	Solubility Parameters at 25 °C	
			Solubility (mg/mL)	Folds Improved
Water	-	-	0.1 ± 0.03	-
PM βCD (1:1)	89.1 ± 2.4	68.3 ± 2.1	0.8 ± 0.3	8
PM HP-βCD (1:1)	92.9 ± 1.7	71.5 ± 1.8	1.2 ± 0.1	12
IR βCD (1:1)	93.6 ± 1.2	75.2 ± 2.3	1.8 ± 0.5	18
IR βCD (1:2.5)	92.3 ± 2.5	85.2 ± 1.7	2.3 ± 0.3	23
IR βCD (1:4)	96.6 ± 3.1	93.9 ± 2.2	3.4 ± 0.4	34
IR HP-βCD (1:1)	96.2 ± 1.9	80.6 ± 2.2	2.5 ± 0.3	25
IR HP-βCD (1:2.5)	95.3 ± 2.1	86.2 ± 1.7	3.2 ± 0.6	32
IR HP-βCD (1:4)	98.2 ± 3.0	95.6 ± 3.1	4.8 ± 0.8	48

**Table 2:** Dissolution parameters of irbesartan from irbesartan solution, PMs, IR-βCD, and IR-HP-βCD inclusion complexes (mean ± SD, *n* = 4).

Formulation	Dissolution Parameter						
	Q_15_	Q_60_	DE_15_	DE_60_	MDT	MDR	IDR
IR *	7.6	15.3	12.5	21.1	24.2	0.2	1.2
PM-βCD	27.7	40.7	21.9	31.2	23.0	0.5	1.8
IR-βCD (1:4)	68.7	76.2	28.4	59.4	15.5	1.3	4.4
PM HP-βCD	29.9	43.9	12.5	52.5	22.2	1.2	1.9
IR-HP-βCD (1:4)	76.9	94.4	35.3	72.4	13.4	1.6	5.1

* IR–Irbesartan; PM-βCD and PM-HP-βCD–a physical mixture of irbesartan with βCD and HP-βCD, respectively.

**Table 3:** Composition of irbesartan and irbesartan-HPβCD complexed solid lipid nanoparticle formulations.

Composition	IR-SLN1	IR-HP-βCD-SLN1	IR-SLN2	IR-HP-βCD-SLN2
Irbesartan (mg)	10	-	10	-
Irbesartan + HPβCD (mg) *	-	52.6	-	52.6
Glyceryl monostearate (mg)	200	200	-	-
Dyanasn 112 (mg)	-	-	200	200
Soyalecithin	200	200	200	200
Poloxamer 188 (mg)	150	150	150	150
Double distilled water up to (mL)	10	10	10	10

* 52.6 mg of the inclusion complex is equivalent to 10 mg of IR.

**Table 4:** Physicochemical characteristics of IR-SLNs and IR-HP-βCD-SLNs (mean ± SD, *n* = 3).

Formulation	Size (nm)	PDI	ZP (mV)	Assay (%)	EE (%)
IR-SLN1	300.7 ± 4.8	0.29 ± 0.08	−20.5 ± 4.1	99.2 ± 1.4	89.1 ± 2.8
IR-HP-βCD-SLN1	339.9 ± 8.3	0.28 ± 0.02	−22.6 ± 3.4	98.4 ± 1.8	86.9 ± 1.8
IR-SLN2	240.2 ± 6.7	0.19 ± 0.05	−29.4 ± 4.8	98.7 ± 1.5	90.6 ± 4.3
IR-HP-βCD-SLN2	257.6 ± 5.1	0.21 ± 0.03	−30.5 ± 4.1	99.8 ± 2.5	93.7 ± 2.5

**Table 5:** Effect of storage at room temperature on physicochemical characteristics of the optimized SLN formulations (mean ± SD, *n* = 3).

Day	IR-HP-βCD-SLN2					IR-SLN2				
	Size (nm)	PDI	ZP (mV)	Assay (%)	EE (%)	Size (nm)	PDI	ZP (mV)	Assay (%)	EE (%)
1	251.4 ± 7.6	0.21 ± 0.03	−30.5 ± 4.1	99.8 ± 2.5	92.4 ± 1.6	234.8 ± 7.2	0.20 ± 0.02	−29.1 ± 3.1	97.9 ± 1.9	91.1 ± 2.5
30	261.5 ± 3.9	0.23 ± 0.05	−28.6 ± 2.0	97.6 ± 1.7	90.7 ± 2.2	244.3 ± 5.9	0.21 ± 0.04	−28.2 ± 2.7	98.3 ± 2.1	90.2 ± 1.9
60	276.6 ± 5.8	0.23 ± 0.06	−27.9 ± 2.9	96.9 ± 2.6	90.1 ± 1.9	256.3 ± 8.6	0.22 ± 0.05	−29.6 ± 4.4	96.8 ± 1.5	88.6 ± 2.2

**Table 6:** Pre- and post-lyophilization effect on physicochemical characteristics of the optimized IR-SLN and IR-CD-SLN formulation (mean ± SD, *n* = 3).

Formulation	Pre-lyophilization					Post-lyophilization				
	Size (nm)	PDI	ZP (mV)	Assay (%)	EE (%)	Size (nm)	PDI	ZP (mV)	Assay (%)	EE (%)
IR-HP-βCD-SLN2	270.3 ± 8.6	0.24 ± 0.05	−28.9 ± 2.7	98.1 ± 1.6	91.6 ± 1.9	465.7 ± 10.5	0.43 ± 0.06	−26.5 ± 1.3	97.6 ± 2.1	92.3 ± 1.6
IR-SLN2	251.3 ± 4.9	0.21 ± 0.02	−31.9 ± 1.8	99.3 ± 2.0	89.6 ± 1.7	410.6 ± 11.9	0.23 ± 0.06	−29.3 ± 2.3	97.6 ± 2.7	88.9 ± 2.1

## Data Availability

Data provided upon request, if needed.
